# Clinical parameters obtained during tear film examination in domestic rabbits

**DOI:** 10.1186/s12917-022-03492-1

**Published:** 2022-11-12

**Authors:** Francesca Corsi, Kevin Arteaga, Flavia Corsi, Marco Masi, Alexia Cattaneo, Paolo Selleri, Manuela Crasta, Claudio Peruccio, Adolfo Guandalini

**Affiliations:** 1 Centro Veterinario Specialistico, Via Sandro Giovannini 51-53, 00137 Rome, Italy; 2 Clinica Veterinaria Oculistica Visionvet, Via Antonio Marzocchi, 6, 40017, San Giovanni in Persiceto, Bologna, Italy; 3 Institute of Molecular Biotechnology of the Austrian Academy of Sciences (IMBA), Vienna BioCenter (VBC), Dr. Bohr-Gasse 3, 1030 Vienna, Austria; 4 Centro Veterinario Torinese (CVT), Lungo Dora Colletta 147, 10153 Torino, Italy

**Keywords:** Rabbits, Tear film, Schirmer tear test-1, Interferometry, Meibography, Tear meniscus, Osmolarity

## Abstract

**Background:**

One of the contributing factors to ocular surface health is a stable precorneal tear film. Considering the increasing interest in rabbits as pets and the limited literature available on domestic rabbit tearing, the aim of this study was to establish normative data for examination of the tear film in domestic rabbits.

**Results:**

The study included 75 client-owned domestic Holland Lop rabbits (150 eyes). The following examinations were performed in each eye: Schirmer tear test-1, tear osmometry, interferometry, tear meniscus height measurement and meibography (quantifying meibomian gland loss as a percentage). The resulting median (95% central range) values were 10.0 (5.0–17.3) mm/min for the Schirmer tear test-1, 345.0 (280.5–376.1) mOsm/L for tear osmolarity, grade 2 (1–4) of interferometry, 0.28 (0.20–0.47) mm for tear meniscus height and 0.0 (0.0–67.6) % meibomian gland loss. A significant association was found between tear osmolarity and age, with an estimated decrease of − 4.0 mOsm/L with each additional year of age (*p* < 0.001). The distributions of interferometry grades were significantly different between males and females (*p* < 0.001), with grade 1 and grade 2 being the most frequent in females and males, respectively. A weak negative correlation was also observed between interferometry grade and the percentage of meibomian gland loss (*r* = − 0.22, *p* = 0.006).

**Conclusions:**

This is an original study that documents extensive tear film parameters in healthy Holland Lop rabbits. The results can be used as normative data for the examination of the tear film in this lagomorph breed.

## Introduction

One of the contributing factors to ocular surface health is a stable precorneal tear film (TF). Qualitative or quantitative modification of the precorneal TF can induce discomfort, corneal epithelium drying, corneal ulceration and an increased prevalence of infectious diseases [1]. The precorneal TF consists of a superficial lipid layer (LL) and an underlying mucoaqueous layer, which constitutes most of the volume of the tears and interacts directly with the glycocalyx of the epithelium via membrane-spanning mucins [2].

Currently, in veterinary medicine, several methods are used to evaluate the quantity and quality of the TF. These include the Schirmer Tear Test (STT), TF osmolarity measurement, interferometry, tear meniscus height (TMH) measurement and meibography.

Considering the relatively high incidence of ocular surface diseases in rabbits [3], several reports have evaluated lacrimal parameters in this species [4–9].

Tear film osmolarity (TFO) is primarily related to the presence of electrolytes in the TF [10]. At present, TFO measurement is considered to be an important parameter in the diagnosis of dry eye in clinical medicine [11]. In the past decade, several studies on osmolarity have been performed in different animal species, such as dogs [10, 12–15], cats [16], horses [17] and rabbits [18]; despite this, it is still not widely adopted as a routine diagnostic test in veterinary medicine [12].

Lipids, which are the main components of the LL, play a significant role in stabilizing the TF and preventing evaporation of the aqueous phase [2, 19]. The TF-LL can be visually analyzed by using tear interferometry. This technique allows evaluation of the behavior of the LL and estimation of its thickness (LLT) based on the interference colors [20]. The results of the study by Arita et al. (2016) [21] showed that interferometric color and fringe patterns were indicative of the balance between the aqueous and LL of the TF, providing information on both the quantity and the quality of the LL and thus allowing the identification of subtypes of dry eye [21].

In veterinary medicine, the lacrimal meniscus is usually measured with the strip meniscometry technique, and this method has been documented in dogs, cats, rabbits and wild animals [22–25]. Within the last few years, SBM Sistemi developed the Ocular Surface Analyzer-Vet (OSA-VET), a portable instrument for veterinary use to help clinicians perform detailed ocular surface (OS) examinations and researchers investigate OS disorders. Measuring the TMH with the OSA-VET instrument is therefore a new technique available to estimate tear volume. The evaluation is based on the interferometric reflection pattern in the space between the lower eyelid and the cornea [26].

Lipids, which constitute the LL, are secreted by meibomian glands (MGs). The only method to visualize MG morphology in vivo is meibography [27]. This can be performed using contact or noncontact techniques. Currently, the noninvasive approach, first described by Reiko Arita in 2008 [28], is the most widely used [27]. With regard to meibography, in veterinary medicine, there is not sufficient available documentation. Rabbits have been studied in main human medicine research projects [29–31], while canine studies have been conducted on meibomian gland disorder (MGD) associated with keratoconjunctivitis, ocular surface disorder (OSD) and sebaceous adenitis [19, 32, 33].

Although the TF in rabbits has already been evaluated in a few studies reported above, they were mostly used as laboratory animals, and the characterization of the TF in Holland Lop rabbits, one of the most common breeds among domestic rabbits, is completely lacking. Considering the increasing prevalence of rabbits as a pets [34] and, consequently, the increasing frequency of rabbits in veterinary practice and the extensive use of rabbits as animal models for ocular diseases in humans, the aim of this study was to estimate normative values of the STT-1, osmolarity, interferometry, TMH and meibography that can be used as reference for examination of the TF in this breed.

## Results

The median age of the studied rabbits was 2 years (range 0.06–11 years), and the female to male sex distribution of the rabbits examined was 52% (*n* = 39) to 48% (*n* = 36). Physical examination was unremarkable in all subjects, although systemic disease could not be completely ruled out given the lack of bloodwork. All rabbits were confirmed to be free of any ocular disease.

All 150 eyes were subjected to the STT-1, interferometry, measurement of the TMH and meibography, while TFO measurement was performed in only 100 eyes of 50 rabbits that presented to the Centro Veterinario Specialistico in Rome.

### Diagnostic test findings

The median (95% central range) values were 10.0 (5.0–17.3) mm/min for the STT-1, 345.0 (280.5–376.1) mOsm/L for TFO measurement, grade 2 (1–4) for interferometry, 0.28 (0.20–0.47) mm for TMH measurement and 0.0 (0.0–67.6) % meibomian gland loss for meibography (Table 1).

**Table 1:** Descriptive statistics of the study population

	Median	95% range	Range (min - max)	Mean ± SD
**Age (years)**	2.00	0.06–9.28	0.06–11.00	2.99 ± 2.83
**STT-1 (mm/min)**	10.0	5.0–17.3	3.0–20.0	9.9 ± 3.4
**TFO (mOsm/L)**	345.0	280.5–376.1	275.0–379.0	337.5 ± 27.1
**Grade of interferometry**	2	1–4	1–4	2 ± 1
**TMH (mm)**	0.28	0.20–0.47	0.16–0.58	0.29 ± 0.07
**Meibomian gland loss (%)**	0.0	0.0–67.6	0.0–90.0	11.2 ± 19.4

Values of STT-1, grade of interferometry, TMH and meibomiam gland loss were calculated on the whole study population (*n* = 150 eyes). Values of TFO were calculated on the subset of individuals visited at Centro Veterinario Specialistico in Rome (*n* = 100 eyes)

*SD* standard deviation

The most frequent grades of interferometry were grade 2 in 56 eyes (37%), followed by grade 1 in 40 eyes (27%), grade 3 in 36 eyes (24%), grade 4 in 18 eyes (12%) and grade 0 in 0 eyes (0%).

No significant differences were found between the distribution of values in the right and left eyes for all the performed tests (STT-1: *p* = 0.6; TFO: *p* = 0.7; TMH: *p* = 0.6; % meibomian gland loss: *p* = 0.4; interferometry: *p* = 0.7).

### Associations of test parameters with age and sex

A significant association between age and TFO values was found, with an estimated TFO decrease of − 4.0 mOsm/L (95% CI = -5.9 – − 2.0; *p* < 0.001) with each additional year of age (Table 2). A weak significant association between age and the STT-1 value was also found, with an estimated STT-1 increase of 0.2 mm/min (95% CI = 0.0–0.4; *p* = 0.02) with each additional year of age (Table 2). However, due to the negligible estimated increase, the wide 95% CI and the *p* value at the boundary of significance, the authors do not consider this latter association to be clinically relevant. No significant association was found between age and grade of interferometry (*p* = 0.5), TMH (*p* = 0.1) or meibography (*p* = 0.6) (Table 2).

**Table 2:** Statistical association between independent variables (age, sex) and diagnostic tests (STT-1, TFO, interferometry, TMH, meibography)

Independent variable	***b*** coefficient	95% CI	***p***-value
**STT-1 (mm/min)**			
**Age**	0.2	0.0–0.4	0.02*
**Sex**	−0.1	−1.2 – 1.0	0.8
**TFO (mOsm/L)**			
**Age**	−4.0	−5.9 – −2.0	3 × 10^− 4^ ***
**Sex**	−10.4	−21.5 – 0.7	0.08
**Interferometry (grades)**			
**Age**	0.1	−0.2 – 0.4	0.5
**TMH (mm)**			
**Age**	3 × 10^−3^	-9 × 10^−4^ – 7 × 10^− 3^	0.1
**Sex**	1 × 10^−5^	−0.02 – 0.02	1.0
**Meibography (% of meibomian gland loss)**			
**Age**	0.0	−0.1 – 0.2	0.6
**Sex**	0.3	−0.7 – 1.3	0.5

**Fig. 1:**
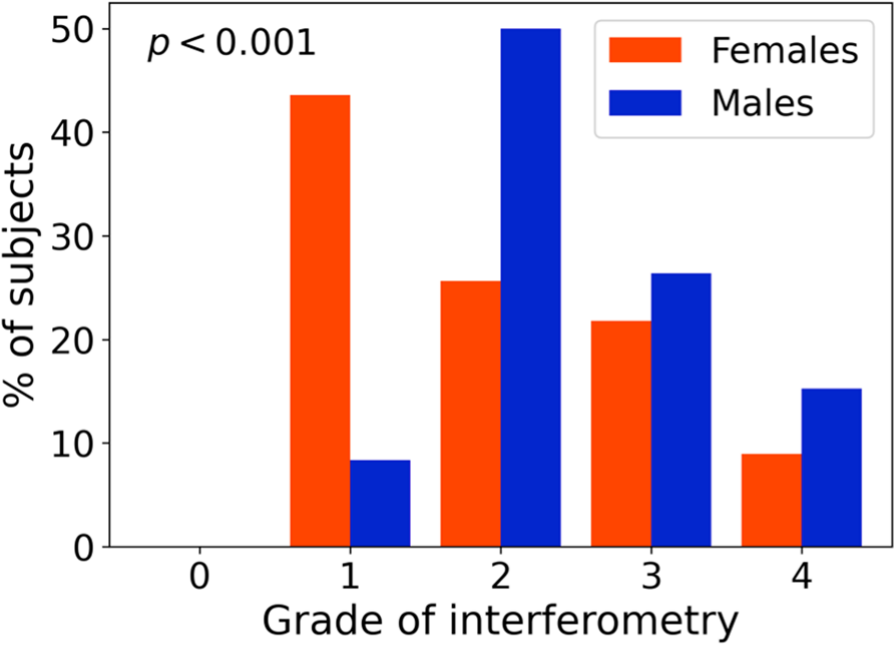
Distribution of sex by grade of interferometry

Statistical associations between independent variables (age, sex) and STT-1, grade of interferometry, TMH and meibomiam gland loss were calculated on the whole study population (n = 150 eyes). Statistical associations between independent variables (age, sex) and TFO were calculated on the subset of individuals visited at Centro Veterinario Specialistico in Rome (n = 100 eyes). Only regression values between age and grade of interferometry are reported in this table, since the frequency distributions of grades of interferometry by sex were evaluated using the Mann–Whitney U test and are reported as a histogram in Fig. 1. Regression coefficients (*b*) were adjusted by sex and age

*CI* confidence interval

Significance codes: * = *p*-value < 0.05; ** = p-value < 0.01; *** = p-value < 0.001

With regard to sex, the distributions of grades of interferometry were significantly different between female and male subjects (*p* < 0.001) (Fig. 1). No other significant association was found between sex and the STT-1 (*p* = 0.8), TFO (*p* = 0.08), TMH (*p* = 1.0) or meibography (*p* = 0.5) value (Table 2).

### Correlations among test parameters

A weak negative correlation was found between the grade of interferometry and the percentage of meibomian gland loss detected by meibography (*r* = − 0.22, *p* = 0.006). No other statistically significant correlations were observed between the STT-1 results, TFO, grade of interferometry, TMH, and meibography (*p* > 0.05).

## Discussion

The TF has multiple functions, including a nutritional function for the cornea and antibacterial, lubricant and refraction functions, contributing to the quality of vision. A considerable number of tests have been developed to evaluate the TF and its optical role [35].

The data and images collected in this study provide a detailed description of the rabbit TF.

In our study population, the mean ± SD of the standard STT-1 was 9.9 ± 3.4 mm/min (Table 1). This value appears to be higher than those obtained in four previous studies carried out in rabbits [4–6, 8], where the mean values of the STT-1 were 7.6 ± 2.3 mm/min [4], 5.30 ± 2.96 mm/min [5], 4.85 ± 2.90 mm/min [6], 5.4 ± 1.6 mm/min (English angora) [8], and 4.6 ± 1.2 mm/min (Dutch rabbits) [8]. A factor contributing to this discrepancy may be the breeds (New Zealand [4, 6], English angora and Dutch rabbits [8]) examined in the other studies. Further studies could assess possible relationships between the different STT-1 values reported and breed predisposition to keratoconjunctivitis sicca, as observed in canine species [36, 37]. The STT-1 variations could also be attributed to other factors, such as the emotional state of the subjects during the examination, the Schirmer strips used, and ambient humidity in the test environment [38].

In the present study, a mean TFO value of 337.5 ± 27.1 mOsm/L was observed for Holland Lop rabbits (Table 1); in the study by Brito et al. (2021) [10], using the same I-PEN® VET osmolarity system, the value was 315.27 ± 6.15 mOsm/L in healthy dogs. In the current study, a negative association was observed between age and TFO (Table 2). A similar relationship was reported in dogs [12] and humans [39]. The former may be due to a decrease in aqueous tear production that occurs with age [40], while the latter has been attributed to a predisposition to total body dehydration with age [41]. The TFO results in this study were higher than those obtained in previous studies carried out in rabbits [18, 42]. This is probably due to different tear measurement methods and differences between the instruments used, as also reported in dogs [10, 13]. In both dogs [43] and humans^35,^ a significant correlation between the STT-1 value and osmolarity was highlighted; therefore, in both cases, the use of osmolarity as a reliable indicator of dry eye was hypothesized. A similar relationship was not observed in the present study, although only healthy rabbits were studied, and the results may differ in rabbits with ocular surface pathology.

The interferometry assessment showed that the distributions of grades were different between female and male subjects. The most frequent grade in females was grade 1 (46%), while in males, it was grade 2 (50%), and only 8% of males presented grade 1 in interferometry (Fig. 1). In both human and veterinary medicine^26,^ it has been shown that the meibomian glands are under neural and hormonal control and that circulating androgens are necessary for physiological meibomian gland function [44]. This could explain the different interferometry grade distribution between male and female subjects observed in this study. It is important to highlight that grade 1 on interferometry was associated with ocular surface disorders in dogs [19], while in this study, grade 1 was observed in asymptomatic females, with the highest percentage.

To date, the strip meniscometry method has been the most commonly used technique in veterinary medicine to evaluate the tear meniscus [22–25]. It is the authors’ opinion that the use of the OSA-VET® instrument is less invasive and presumably less stressful for patients than the strip meniscometry test, as it uses images directly obtained from the interferometry exam. The observed TMH results (mean ± SD: 0.29 ± 0.07 mm) (Table 1) can be considered normative data in rabbits; the mean value was somewhat similar to that in cats (0.31 ± 0.09 mm) but lower than that in dogs (0.53 ± 0.11 mm) [26].

The slightly negative correlation between interferometry grade and meibography value was consistent with that of data obtained in the study by Viñas et al. [19], who found a higher risk of MG dysfunction at grade 0 with a thinner LL; in this study, in asymptomatic rabbits, a lower risk was found for grade 2 with a thicker LL.

Interestingly, the overall low interferometry grades observed in rabbits and the paucity of rabbits with MG loss were consistent with the general perception that the rabbit TF is highly stable [45]. Indeed, rabbits generally blink only three or four times an hour [46]. The harderian glands of some vertebrates may produce new lipids that stabilize the TF and protect against dry eye. Lipids in the rabbit’s harderian glands and tears differ from those identified in human meibum and tears. These unique rabbit lipids confer a protective effect against evaporative dry eye disease [47]. In addition, the lacrimal gland in rabbits is a heterogeneous gland that is composed of both serous and mucin-secreting cells, and mucins produced by this gland contribute to the mucin pool on the ocular surface. Moreover, the composition of tears produced by the rabbit’s lacrimal gland may differ depending on the bodily needs and the environment, resulting in differential secretion [48].

The main limitation of this study is the focus on a single breed (Holland Lop) and a relatively young population (median age 2 years); therefore, the results cannot be generalized to other breeds or older subjects. Furthermore, bloodwork was not performed in any subject; therefore, we cannot exclude potential systemic disorders in the study rabbits that could have influenced ocular surface homeostasis. Last, the study did not assess TF breakup time, a common diagnostic test for tear film stability, or other tests, such as basal tearing with the STT-2.

## Conclusions

In the current study, several parameters of the rabbit TF were evaluated. The values reported in Table 1 can be used as normative data for the examination of the TF in this breed. Other pertinent findings included reduced TFO with increasing age, different distributions of interferometry grades between males and females, and a lower interferometry grade with an increasing percentage of MG loss.

## Materials and methods

The present study is in compliance with the principles of the Società di Oftalmologia Veterinaria Italiana (SOVI). Furthermore, ethical approval under Directive 2010/63/EU was deemed unnecessary since the study applied minimally or noninvasive examinations and diagnostic tools, falling under the scope of Article 1 paragraph 5(f) of the same Directive. Informed consent was obtained from all owners.

A total of 75 client-owned domestic Holland Lop rabbits (*n* = 150 eyes) were enrolled in the study. The subjects were selected from rabbits that presented to two different clinics for vaccinations or other preventative care: Centro Veterinario Specialistico in Rome, Italy (*n* = 50 rabbits), and Clinica Veterinaria Oculistica Visionvet in Bologna, Italy (*n* = 25 rabbits).

Prior to inclusion in the study, all rabbits underwent a complete ophthalmic examination. Procedures were performed in the following order: ocular surface staining with fluorescein, applanation tonometry (Tono-Pen®, Reichert, Depew, NY, USA), slit-lamp biomicroscopy (SL-14®, Kowa Company, Sakai, Osaka, Japan) and indirect ophthalmoscopy (Heine, Omega 500). All rabbits were confirmed to be free of any ocular disease.

### Tear film examination

**Fig. 2:**
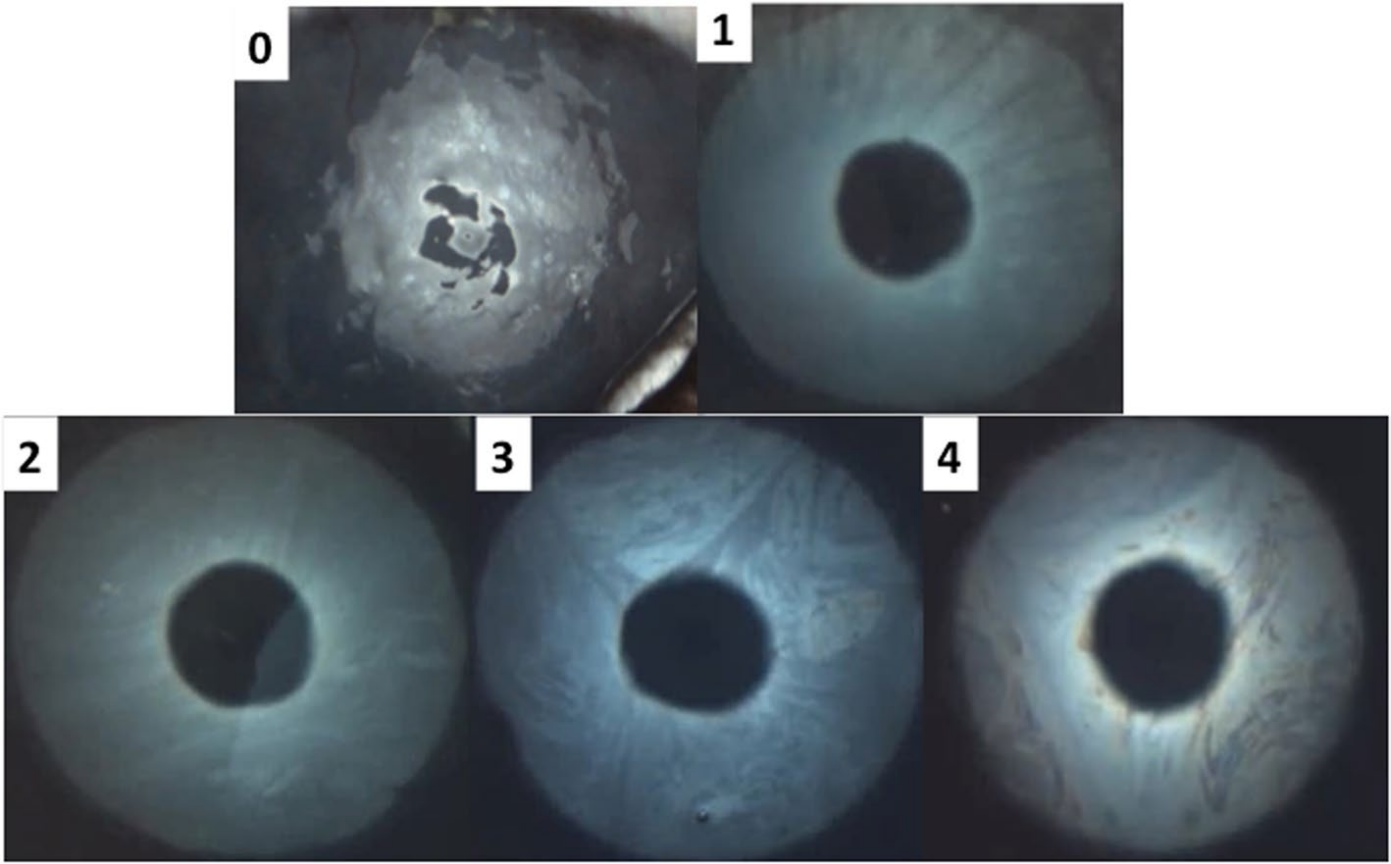
Grading of interferometric patterns in rabbits. Grade **0** corresponds to almost complete absence of the aqueous phase (a dog’s interferometry published with copyright permission [19]); grade **1** (15–30 nm of thickness) corresponds to a faintly visible homogeneous meshwork pattern; grade **2** (31–60 nm of thickness) corresponds to a more compact meshwork pattern with gray waves and occasional colored shades; grade **3** (61–100 nm of thickness) corresponds to a meshwork with waves and interference fringes with some colors noted; and grade **4** (more than 100 nm of thickness) corresponds to waves with many colors [19]

**Fig. 3:**
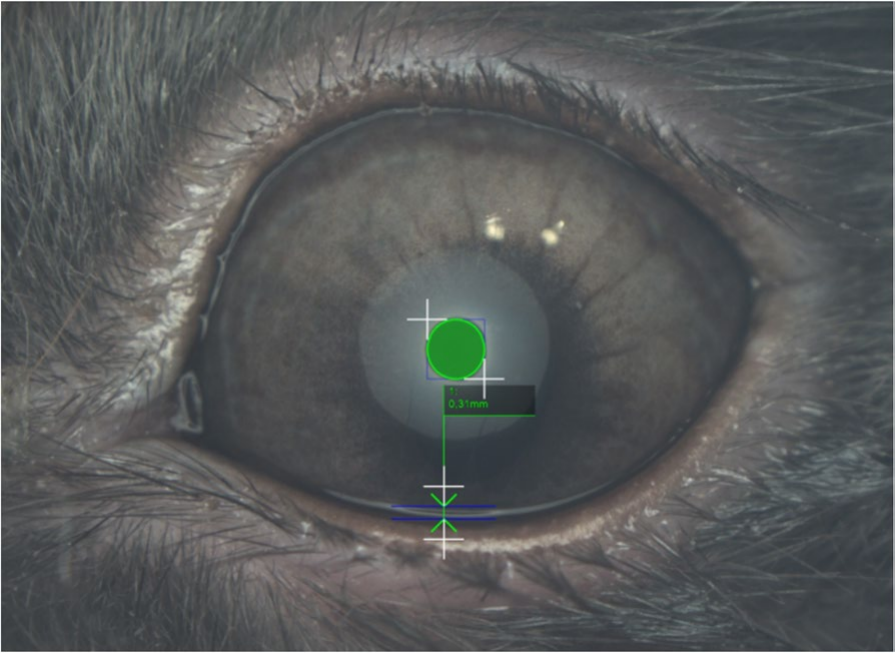
Tear Meniscus Height evaluation with the OSA-VET. Tear Meniscus Height evaluation with the OSA-VET is based on the interferometric reflection pattern in the space between the lower eyelid and the cornea

**Fig. 4:**
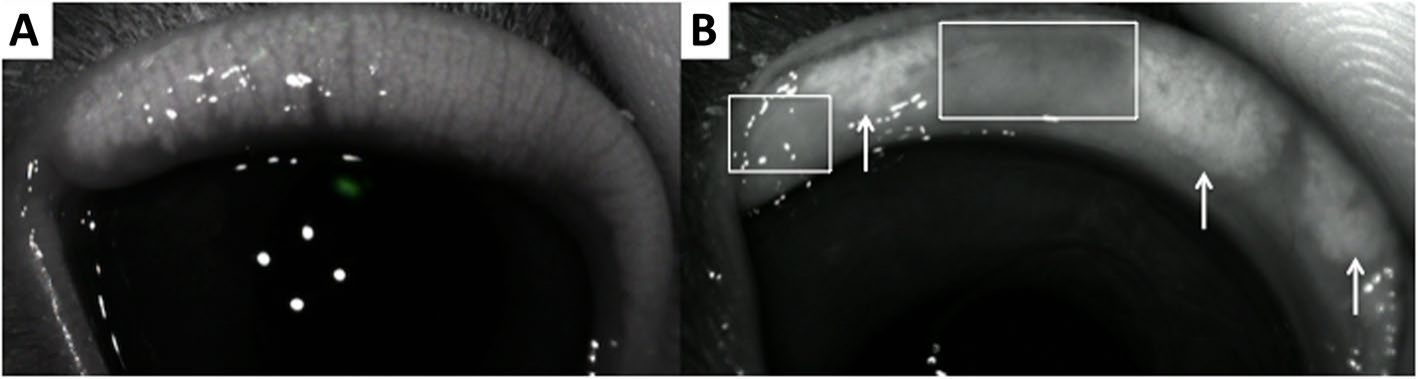
Noncontact meibography. **A** Normal meibomian glands in an upper eyelid examined with noncontact meibography. **B** An upper eyelid with visible areas of meibomian gland loss (rectangles) and loss of normal architecture of the remaining meibomian glands (arrows)

To avoid excessive interobserver differences, all TF examinations were carried out by the same two operators (ECVO residents: FC, KA) helped by two veterinary technicians with expertise in this field under the supervision of two board-certified veterinary ophthalmologists (AG, MC). Basal and reflex tear production was measured with the Schirmer tear test-1 (STT-1) in all rabbits. A standardized strip of filter paper (Merck) was placed in the conjunctival fornix in the middle third of the lower eyelid of each eye to measure wetting in millimeters over a 1 minute period (mm/min). Thirty minutes later, TFO was performed in both eyes of all rabbits that presented to the first clinic (*n* = 100 eyes); the measurements were taken using the I-PEN® VET Tear Osmolarity System (I-Med Pharma), and samples were collected by placing a single-use sensor in the inferior tear meniscus near the lateral canthus. After 2 seconds of contact, the instrument displayed a quantitative numerical value in units of mOsms/L. Thirty minutes later, interferometry, TMH measurement and meibography were carried out in both eyes of all rabbits using an ocular surface analyzer (OSA-VET®, SBM Sistemi, Torino, Italy), a handheld device that utilizes infrared and white LED lights. First, an interferometry exam was performed consisting of 60 seconds of video that was recorded in each eye. The images were then used to estimate the TF-LL pattern, with each revealing a specific thickness of the TF-LL. The patterns were classified according to a five frame-grading scale [49] as follows: “Grade 0 corresponds to almost complete absence of the aqueous phase; grade 1 (15–30 nm) corresponds to faintly visible homogeneous meshwork pattern; grade 2 (31–60 nm) involves a more compact meshwork pattern with gray waves and occasional colored shades that can be observed; grade 3 (61–100 nm) corresponds to a meshwork with waves and interference fringes with some colors are noted; and grade 4 (more than 100 nm) corresponds to waves with many colors” [19] (Fig. 2). The TMH was measured using software that processed the selected interferometric image 3 seconds after blinking. TMH evaluation with the OSA-VET® is based on the interferometric reflection pattern in the space between the lower eyelid and the cornea [26] (Fig. 3). Meibography was conducted as the last examination. Due to the difficulty in everting the lower eyelid in this species, meibography was performed on only the upper eyelid of each eye. To minimize image reflections, each exam was performed in a dark room without lighting, and images were obtained for the entire area of the upper eyelid for both eyes. Using the instrument’s software, it was possible to calculate the percentage of the MG-loss area (Fig. 4).

## Statistical analysis

Statistical analysis was performed with R software (version 3.6.3). Normality of the data was assessed using the Shapiro–Wilk test. Since the data were not normally distributed (*p* < 0.01), the results are summarized as the median and 95% central range (2.5th - 97.5th percentiles) and the mean and standard deviation (SD) for comparisons with the results of previous studies. Absolute frequencies and percentages are used to describe categorical (sex) and ordinal (interferometry grades) variables. The distributions of the STT-1, TFO, interferometry, TMH and meibography values for the right and left eyes were compared using the Wilcoxon Signed-Rank Test. The frequency distributions of grades of interferometry by sex were evaluated using the Mann–Whitney U test.

Associations between independent (age and sex) and dependent variables (STT-1, TFO, TMH and percentage of meibomian gland loss) and age and grade of interferometry were assessed using mixed-effects models, which allowed us to account for intraclass correlations, i.e., between the eyes of the same subject and among eyes tested by the same operator. Specifically, linear mixed-effects models were used to analyze STT-1, TFO and TMH continuous values; a generalized mixed-effects model with a negative binomial distribution was used to analyze the percentage of MG loss; an ordinal mixed-effects model was used to analyze the association between age and grade of interferometry. Regression coefficients (*b*) were adjusted after controlling for sex and age and are reported with their 95% confidence intervals (CIs) and *p* values (*p*) to assess statistically significant associations. The Spearman correlation coefficient (*r*) was calculated to assess correlations between pairs of diagnostic tests. *p* values < 0.05 were considered statistically significant.

## Data Availability

The datasets used and/or analyzed during the current study available from the corresponding author on reasonable request.

## Abbreviations

TF: Tear film
STT-1: Schirmer tear test-1
TFO: Tear film osmolarity
TMH: Tear meniscus height
LL: Lipid layer
LLT: Lipid layer thickness
N.I.B.U.T.: Non invasive break up time
MGs: Meibomian glands
MGD: Meibomian gland disorder
OSD: Ocular surface disorder
